# Endurance exercise preserves physical function in adult and older male C57BL/6 mice: high intensity interval training (HIIT) *versus* voluntary wheel running (VWR)

**DOI:** 10.3389/fragi.2024.1356954

**Published:** 2024-03-07

**Authors:** Megan L. Pajski, Chris Byrd, Nainika Nandigama, Emily Seguin, Anna Seguin, Alyssa Fennell, Ted G. Graber

**Affiliations:** ^1^ Department of Physical Therapy, East Carolina University, Greenville, NC, United States; ^2^ Department of Public Health, East Carolina University, Greenville, NC, United States; ^3^ Department of Kinesiology, East Carolina University, Greenville, NC, United States; ^4^ Department of Physiology, East Carolina University, Greenville, NC, United States; ^5^ East Carolina Obesity and Diabetes Institute, East Carolina University, Greenville, NC, United States

**Keywords:** sarcopenia, exercise, mice, endurance training, aging

## Abstract

Exercise has been shown to improve physical function, mitigate aspects of chronic disease and to potentially alter the trajectory of age-related onset of frailty and sarcopenia. Reliable and valid preclinical models are necessary to elucidate the underlying mechanisms at the intersection of age, exercise, and functional decline. The purpose of this study was to compare, head to head, the effects of two common pre-clinical models of endurance exercise: high intensity interval training (HIIT) and voluntary wheel running (VWR). The hypothesis was that a prescribed and regimented exercise program, HIIT, would prove to be a superior training method to unregulated voluntary exercise, VWR. To investigate this hypothesis, we evaluated adult (n = 24, designated 10 m, aged 6 months at the beginning of the study, 10 months at its completion) and older adult (n = 18, designated 26 m, aging from 22 months to 26 months over the course of the study) C57BL/6 male mice. These mice were randomly assigned (with selection criteria) to a 13-week program of voluntary wheel running (VWR), high intensity interval training (HIIT), or sedentary control (SED). The functional aptitude of each mouse was determined pre- and post-training using our composite CFAB (comprehensive functional assessment battery) scoring system consisting of voluntary wheel running (volitional exercise and activity rate), treadmill (endurance), rotarod (overall motor function), grip meter (forelimb strength), and inverted cling (whole body strength/endurance). To measure sarcopenia, we tracked body mass, body composition (with EchoMRI), plantar flexor torque (in 10 m), and measured muscle wet mass post-training. Overall, adult CFAB scores decreased while body mass and percent body fat increased as they matured; however, exercise significantly mitigated the changes (*p* < 0.05) compared to SED. Older adults demonstrated preservation of function (CFAB) and reduced body fat (*p* < 0.05) compared to SED. To conclude, both types of exercise maintained physical function equally in older mice.

## Introduction

Exercise, and increasing physical activity in general, has been well-described as an intervention with efficacy to mitigate a host of chronic diseases and age-related syndromes (Pedersen and Saltin, 2006; Landi et al., 2014). For example, adequate physical activity, such as the minimums (e.g., 150–300 min of moderate activity and 2 day/week of strength training) described by the U.S. Department of Health and Human Services (2023), has been shown to prevent/delay the onset of diabetes, cardiovascular disease, cancer, and metabolic syndrome, including obesity (Pedersen and Saltin, 2006; Medeiros et al., 2008; Garcia-Valles et al., 2013). Exercise also plays a role in maintenance of cognitive function and muting the trajectory of cognitive decline (Klusmann et al., 2010; Theou et al., 2011; Tarazona-Santabalbina et al., 2016).

Sarcopenia is traditionally referred to as the “age-related loss of muscle mass and strength or function” though there are varying definitions among clinicians and researchers (Evans et al., 2024). One clinical definition is “…probable sarcopenia is identified by Criterion 1. Diagnosis is confirmed by additional documentation of Criterion 2. If Criteria 1, 2 and 3 are all met, sarcopenia is considered severe. 1 Low muscle strength, 2 Low muscle quantity or quality, 3 Low physical performance…” (Cruz-Jentoft et al., 2019). Sarcopenia is also now recognized as a disease in the ICD-10 (Vellas et al., 2018). After the age of fifty, muscle mass decreases by 1%–2% annually while muscular strength can decrease 12%–15% every 10 years (Larsson, 1983; Quittan, 2016). Reduced physical function is a major consequence of sarcopenia leading decreased ability to perform activities of daily living (ADLs), loss of independence, onset of disability and frailty, and increased mortality (Reeves et al., 2004; Marzetti et al., 2017). Low muscle mass correlates to weakness in older adults (Newman et al., 2003), while weakness has a strong positive correlation with decreased function (Schaap et al., 2013) and mobility (Visser et al., 2005). Rodent models demonstrate similar sarcopenic outcomes to those mentioned above in humans (Parks et al., 2012; Graber et al., 2013; Liu et al., 2014; Graber et al., 2021). Exercise, however, can help mitigate frailty and preserve physical function in both humans and mice (Koster et al., 2012; Reeves et al., 2004; Graber et al., 2015; Medeiros et al., 2008; Seldeen et al., 2019).

The large-scale MoTrPAC (molecular transducers of physical activity) study financed by the NIH common fund has been initiated to learn more about the mechanisms by which exercise improves health, in humans and rats (Sanford et al., 2020). In humans, endurance exercise programs produce improvements in body composition, endurance, physical function, and to a lesser degree, strength (Wanderley et al., 2015; Henderson et al., 2017; Villareal et al., 2017; Yoo et al., 2018). In contrast, resistance training (e.g., weight lifting) in humans primarily improves strength and muscle mass, with less improvement in aerobic capacity than endurance training (Phillips SM, 2007). Similar results have been observed in mouse models of exercise (Graber et al., 2015; Graber et al., 2019a; Seldeen et al., 2019).

In this study we determined the efficacy of two mouse endurance exercise models, voluntary wheel running (VWR) and high intensity interval training (HIIT), to preserve or improve physical function in older adult and adult male C57BL/6 mice (Liu et al., 2014; Graber et al., 2015; Seldeen et al., 2018; Seldeen et al., 2019). In VWR, the mice choose to exercise at will on a running wheel—which would be similar to a human study where participants are given a pedometer, and their activity rate is tracked without any directed exercise goals. HIIT is a prescribed program of progressively increasing challenge where intervals of intense activity (in this case sprinting on a treadmill) are alternated with a lower intensity (walking on the treadmill) recovery period. In mice, HIIT is designed similarly to how humans would perform the same exercise on a treadmill (Seldeen et al., 2018; Seldeen et al., 2019). We measured exercise capacity and physical function with our previously designed composite scoring system, CFAB (comprehensive functional assessment battery), currently validated only in male C57BL/6 mice (at 6 m, months old, 24 m, and 28 m) and not female mice (Graber et al., 2021). CFAB, the primary outcome measure of the current study, is composed of 5 well-validated determinants: wheel running (volitional activity rate), grip meter (forelimb strength), inverted cling (four-limb strength/endurance), rotarod (overall motor function), and treadmill max-speed test (aerobic capacity) (Graber et al., 2021). *A priori*, we hypothesized that in comparison to VWR, the prescribed, personalized, and regimented HIIT protocol would provide a greater overall improvement in physical function and exercise capacity. To test this hypothesis, we randomized male C57BL/6 mice at two different ages into exercise groups (VWR and HIIT): adults (A) starting training at 6 m (months-old) adults (roughly early to mid-20s human equivalent) and then ending at 10 m (corresponding roughly to early middle age in humans), and older adults (OA) training from 22 m to 26 m (at the study end the mice were roughly equivalent to early 70s in humans). We had two sedentary control groups (SED) that did not exercise: 6m–10 m adults (SEDA) and 24m–28 m (SEDOA). See the online-only Supplemental Discussion for a comparison of human *versus* mouse ages and how we estimate mouse to human month to year calculations. Pre- and post-training, we determined body composition (with MRI), physical function with CFAB, and maximal isometric plantar flexor torque with *in vivo* contractile physiology.

## Methodology

### Animals

We obtained male C57BL/6 mice from the NIH NIA Aging Rodent Colony and from Charles River Laboratory. The mice were treated humanely under protocols approved by East Carolina University IACUC (protocol #p106). The mice were group housed in large static microisolator mouse cages come from Ancare (N40 cage bottom top hi-temp polycarbonate, N40 micro filter top hi-temp polycarbonate, and the N40SS wire lid stainless steel), unless separated for extreme aggression and fighting. They were kept at 22°C with 12-h light/dark cycles and fed/watered *ad libitum* with standard rodent chow. At the start of the study, we had n = 24 adult mice (aged 6 m), n = 18 older adult mice (22 m) and n = 10 older adult mice (24 m). During the study, four adult and four older adult mice died from natural causes or were euthanized by recommendation of the ECU veterinary staff. Sample size (minimum n = 7) was determined using power analysis based upon the CFAB mean (−5.74) and standard deviation, SD (0.44), for 28-month-old mice from our previous work with the second group to have a predicted minimum difference of 15% (−4.879, assuming equal variance) generated effect size of 1.957 with α = 0.05 and power = 95% in a two-tailed independent samples Student’s t-test using the software program G-Power (Faul et al., 2007; Faul et al., 2009; Graber et al., 2021). Furthermore, a power analysis of changes between group CFAB means for a mixed model ANOVA at two time points with three groups (group 1, control, mean as above, and groups 2 and 3, exercise treatments, with predicted 15% equal change of mean = −4.879, and assuming equal standard deviation of 0.44 for all groups) showed a strong effect size of 0.922 with α = 0.029 and power = 97% at n = 7.

### Study design

**FIGURE 1:**
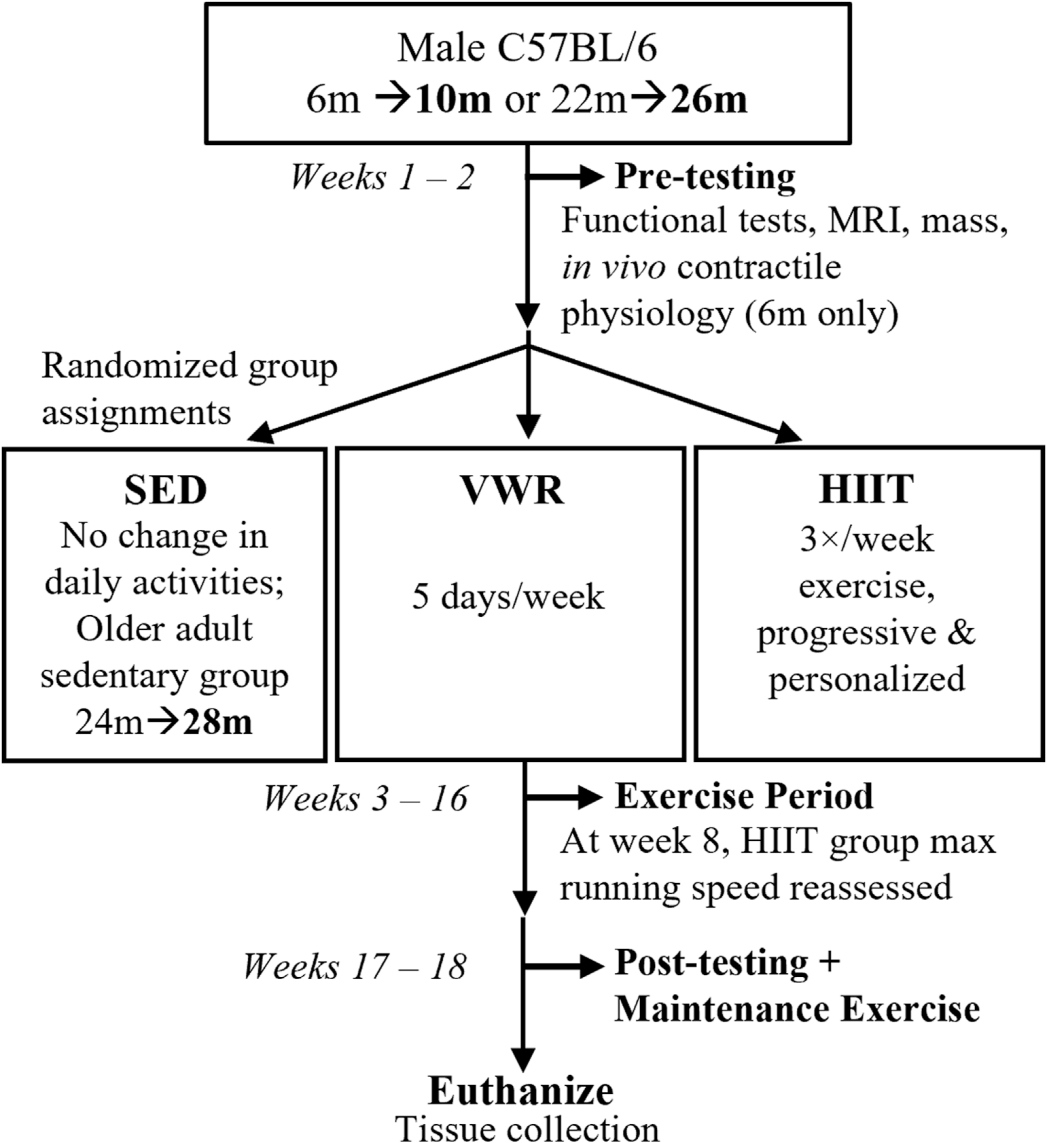
Study Design. Prior to exercise assignments, male C57BL/6 mice aged 6 months, m (10 m at study end, n = 24) or 22 months (26 m at study end, n = 18) were pre-tested for functional abilities (voluntary wheel running or VWR, rotarod, treadmill, grip strength, inverted cling), as well as mass, body composition (MRI, magnetic resonance imaging), and *in vivo* contractile physiology (maximum isometric plantar flexor torque). Afterwards, mice were randomized into volitional exercise/VWR (10 m n = 8, 26 m n = 8), HIIT, high intensity interval training (10 m n = 8, 26 m n = 10), or sedentary controls/SED (10 m n = 8). We had an additional slightly older adult SED group (24 m n = 8). For the next 13 weeks, VWR groups spent 5 days/week singly-housed in cages equipped with running wheels (and 2 days group housed with cage mates), HIIT groups trained 3 days/week, and SED groups remained in home cages. HIIT and SED mice (and VWR mice on the 2 days/week) were group housed. After exercise training, the mice post-tested before being euthanized for tissue collection. Maintenance exercise 2 days/week was provided for HIIT groups between the end of the exercise period and euthanasia.

See Figure 1 for study design schematic. We assessed the mice for physical function and then randomly selected them into groups based on age and exercise type. Age groups were defined as: A, adults (10 m at study completion), and OA, older adults (26 m at study completion for both exercise groups, while the sedentary group was 28 m at study completion). We defined the exercise groups as: VWR, voluntary wheel running, HIIT, high intensity interval training; and SED, sedentary control (no exercise). Due to complications during the Covid-19 pandemic shutdown we added an additional control group of slightly older adult sedentary control mice (n = 10, 24 m at start and 28 m at finish, SEDOA) since we were unable to complete a 26 m SED group because of laboratory lockdowns. Only CFAB and mass were measured in the SEDOA group to determine the effect of 4 months of sedentary behavior on older adult mice. See section on Exercise Training for more details.

We established an exclusion criterion to be selected into the VWR exercise group (<500 revolutions per week on the running wheels during pretesting). This exclusion criteria ensured that mice randomized to VWR would actually exercise voluntarily to at least a minimal degree. Without this exclusion, the VWR group may have had mice with no internal voluntary exercise drive, thus invalidating the results (cannot measure exercise results if the subjects do not exercise!). Mice running below the 500 revolution per week pre-testing threshold were randomized into either HIIT or CON. Similar to successful training durations previously established, the mice exercised for 13 weeks (including a 1-week acclimation period), followed by post-training functional assessment and subsequent tissue collection (Graber et al., 2019a).

### Functional testing

#### Neuromuscular Performance

We determined physical function and exercise capacity using the Comprehensive Functional Assessment Battery (CFAB) similar to as previously described (Graber et al., 2021). CFAB is a composite scoring system measuring overall physical function consisting of five common non-colinear well-validated determinants, including grip meter for fore-limb strength (normalized to body mass), inverted cling for overall strength/endurance (log10 transformation to assure normality), rotarod for overall motor function (coordination, balance, endurance, power production), voluntary wheel running (volitional exercise and activity rate, with endurance (log10 transformation to assure normality), and treadmill running (max speed test for aerobic endurance/capacity and running speed). We administered these tests pre- and post-training. Devices used for each test were: grip meter (Bioseb GT3), treadmill max speed test (Columbus Instruments Exer 3/6Treadmill), rotarod (Panlab LE820 from Harvard Apparatus), VWR (Columbus Instruments Mouse Home Cage Running Wheel), and inverted cling (custom built device consisting of a hinged grid top over a 56 cm tall × 29 cm wide × 27 cm deep plexiglass box with a padded bottom). CFAB was derived for each individual mouse as the sum of the individual standardized scores for each of the five determinants (reference group is the mean and standard deviation of the 6 m male C57BL/6 mouse published in Graber et al., 2021). We then compared the pre- and post-CFAB results to determine the effect of the three conditions (HIIT, VWR, or SED) on functional capacity over the 4 month period. A perfectly average 6 m old mouse would rate as a zero CFAB, a positive score indicates function greater than average, whereas a negative CFAB score indicates function worse than the average 6 m mouse, i.e., the more negative the score, the worse the overall functional performance and exercise capacity of the mouse. Further information on the specific Individual testing procedures are discussed briefly in the Online Supplement Methodology Section and have been previously published in detail (Graber et al., 2013; Graber et al., 2015; Graber et al., 2018; Graber et al., 2019a; Graber et al., 2021).

### Other tests

#### *In vivo* contractile physiology

We measured maximal plantar flexor torque production (1300A: 3-in-1 Whole Animal System–Mouse, Aurora Scientific) in the 10 m HIIT, VWR, and CON, pre- and post-training period. Due to Covid19 restrictions and laboratory limitations we were unable to collect this data for the OA mice. Methods have been published elsewhere (Graber et al., 2019b; Neelakantan et al., 2019; Brightwell et al., 2021; Graber et al., 2021) and see further details in the online supplement. In brief, we anesthetized the mouse using isoflurane (∼3% isoflurane and 1.5 L/m of O_2_, to effect) to remove conscious control of skeletal muscle. We then shaved the lower limb, and the mouse was situated on a heated platform with the tibia parallel to the platform and the femur condyle clamped to prevent shifting of the leg, forming a 90° angle. The foot was set into a footplate attached to a force transducer adjusted to provide minimum resistance and then we found the optimal placing of the needle electrodes to produce a maximum torque twitch in the plantar flexors (gastrocnemius complex) using 2 mA of current. Next, we determined the optimal current needed to produce the maximum isometric twitch by increasing the amperage applied for sequential twitches in sequence (0.5 mA, 1mq, 2 mA, 3 mA, 4 mA, 5 mA…) to find the largest twitch torque prior to decrease or plateau of torque at the minimum needed current (to avoid off target muscle stimulation). Keeping the needles and current set as were determined while finding the maximal twitch torque, we then found maximum isometric tetanic torque (mN*m, milliNewtons multiplied by meters) by constructing a torque-frequency curve from a single pulse, 10 Hz (Hz), 40 Hz, 80 Hz, 100 Hz, 120 Hz, 150 Hz, 180 Hz, 200 Hz, and finally a second single pulse, with a 1 min rest between each stimulus. These contractile values were normalizing to body mass (mN*m/gbm, gbm = grams of body mass).

#### Body Composition

Body mass was measured during *in vivo* contractile physiology, at the time of tissue collection prior to euthanasia, and once a week during the treadmill and running wheel protocols. EchoMRI-700 (Echo Medical Systems) was used to determine body composition (fat percentage, fat%) at pre- and post-training. Due to Covid19 restrictions and laboratory limitations we were unable to collect this data for SEDOA. We thus substituted, for the sake of comparison, body composition data previously collected using DEXA (dual-energy x-ray absorptiometry) on a different cohort (n = 10) of sedentary mice aged between 23 and 27 months that was previously reported (Graber et al., 2019b).

#### Muscle Mass

We collected tissue during non-survival surgery just prior to euthanasia (via exsanguination and removal of the heart). The mice were deeply anesthetized with a ketamine/xylazine mix (144 mg/kg ketamine, 16 mg/kg xylazine, to effect). We then collected the hindlimb muscles, including dorsiflexor (tibialis anterior, TA), plantar flexors (gastrocnemius, GAS; soleus, SOL; plantaris, PLANT), and extensor digitorum longus (EDL) (Charles et al., 2016). We recorded the wet mass of muscles (mg). We then combined the total mass of the collected muscles into a parameter we termed *total muscle mass*.

### Exercise training

The mice were divided into groups as described above: VWR (both ages n = 8, with 7 OA and 6 A surviving to complete the study, but see below), high intensity interval training (HIIT, A n = 8 all completed the study; and OA n = 10, 9 OA completed training), and sedentary control (SED, A, n = 8, 6 completed; OA, n = 10, 8 completed. Three mice were euthanized as instructed by our veterinary staff due to extreme dermatitis and were unable to complete the study (n = 2 A and n = 1 OA), the other mice died of natural causes prior to study completion. One mouse that was originally randomized into the VWRA group refused to exercise and was thus moved to the SEDA group (final count: VWRA n = 5 and SEDA n = 7).

Sedentary mice (SEDA and SEDOA) were group housed in cages with environmental enrichment but did not exercise. Exercise mice trained as follows:

#### VWR

VWR training protocols were the same as VWR functional testing, except mice were singly housed in cages equipped with running wheels for 5 days, followed by a two-day period when mice were returned to social housing for rest and recuperation. Each VWR mouse completed one five-day session per week, with mass and total wheel revolutions recorded at the end of the 5 days (converted to km/day). We tracked weekly VWR exercise volume (measured as work done in units of grams body weight × distance ran).

#### HIIT (prescribed treadmill running)

HIIT protocols were modified from Seldeen and others (2019), differing in that we did not have the mice run at an incline. Using maximum running speed at failure from pre-training treadmill functional testing, HIIT mice were placed in similarly paced groups. The median max speed of each group was used to determine 75%, 80%, and 85% max speed for HIIT intervals. Mice trained three times per week with a two-day rest during weekends. HIIT sessions began with a 1-min 3 m/min warm-up walk, followed by intervals separated by 60 s walk steps (3 m/min). Each interval began with a 30 s acceleration step to running speed (set to 75%, 80%, or 85% max speed), which was maintained for 60 s, before ending with a 30 s deceleration back to walking speed. HIIT sessions ended with a 2-min 3 m/min cool-down walk. Initially, groups trained at lower-intensity, fewer HIIT intervals (three intervals at 75% max speed, or 75-75-75). Over time, interval number and running speed increased according to the abilities of the mice until groups were running 75-80-85-80-75 intervals. This might be considered a typical recommended intensity scheme for HIIT in humans (Mack, 2018; Maillard et al., 2018; Marriott et al., 2021). Halfway through the study, HIIT mice repeated the functional treadmill test with group speeds and assignments changed according to how the maximum running speed of each mouse had improved. We tracked weekly HIIT exercise volume (measured as work done in units of grams body weight x distance ran) and power produced (work/total seconds of exercise session). The HIIT groups were groups housed.

### Statistics and data

Unless otherwise noted, we report the mean plus or minus the standard error of the mean (SE). For analysis, experimental groups were assigned number identifiers and statistical tests were performed by a lab member blind to the group ID meanings. Statistical significance was set at *p* < 0.05, and trends are reported where 0.05 < *p*< 0.10. One-way ANOVA, or Student’s t-test are used to compare means between subjects, as appropriate. We used 3 × 2 × 2 (3 exercise groups, 2 age groups, and 2 time points) mixed model ANOVA, 3 × 2 (3 exercise groups and 2 time points) mixed model ANOVA and paired t-tests for with-in subject changes and to analyze main effects. Our main outcome was the difference in CFAB from pre-to post-training (normally distributed, and tested with Tukeys’s Honest Significant Difference, HSD, posthoc). As we normally do, to minimize type two error from the inherently large variability in CFAB determinants, we used least significant differences (LSD) *post hoc* testing and we report Tukey’s HSD alongside for comparison. If tests had outliers (defined *a priori* as greater than >2 standard deviations from the mean), we analyzed both with and without these outliers and report both, if needed, for clarity (i.e., if the outlier changes the statistical test). In addition, we report effect sizes as appropriate (either Cohen’s d or η^2^ (or partial η^2^) in the Supplementary Tables. We specify the statistical test used for each set of data in the results and figures/tables. We report effect size, skew, kurtosis, and results of normality tests in Supplementary Tables S1−S4. Contrary to previous work the VWR measurement in km/day severely violated normality, but was normally distributed with a log10 transformation. We thus used the log10 transformation to construct CFAB. Non-parametric tests were used where violations of normality occurred (e.g., the Kruskal-Wallace 1-Way ANOVA or Wilcoxon Signed Rank Test, see Supplementary Tables S1−S4 for more details). We used SPSS v28 and v29 (IBM) for statistical analyses.

## Results

### Physical function (CFAB): improved, maintained or loss mitigated with exercise

**FIGURE 2:**
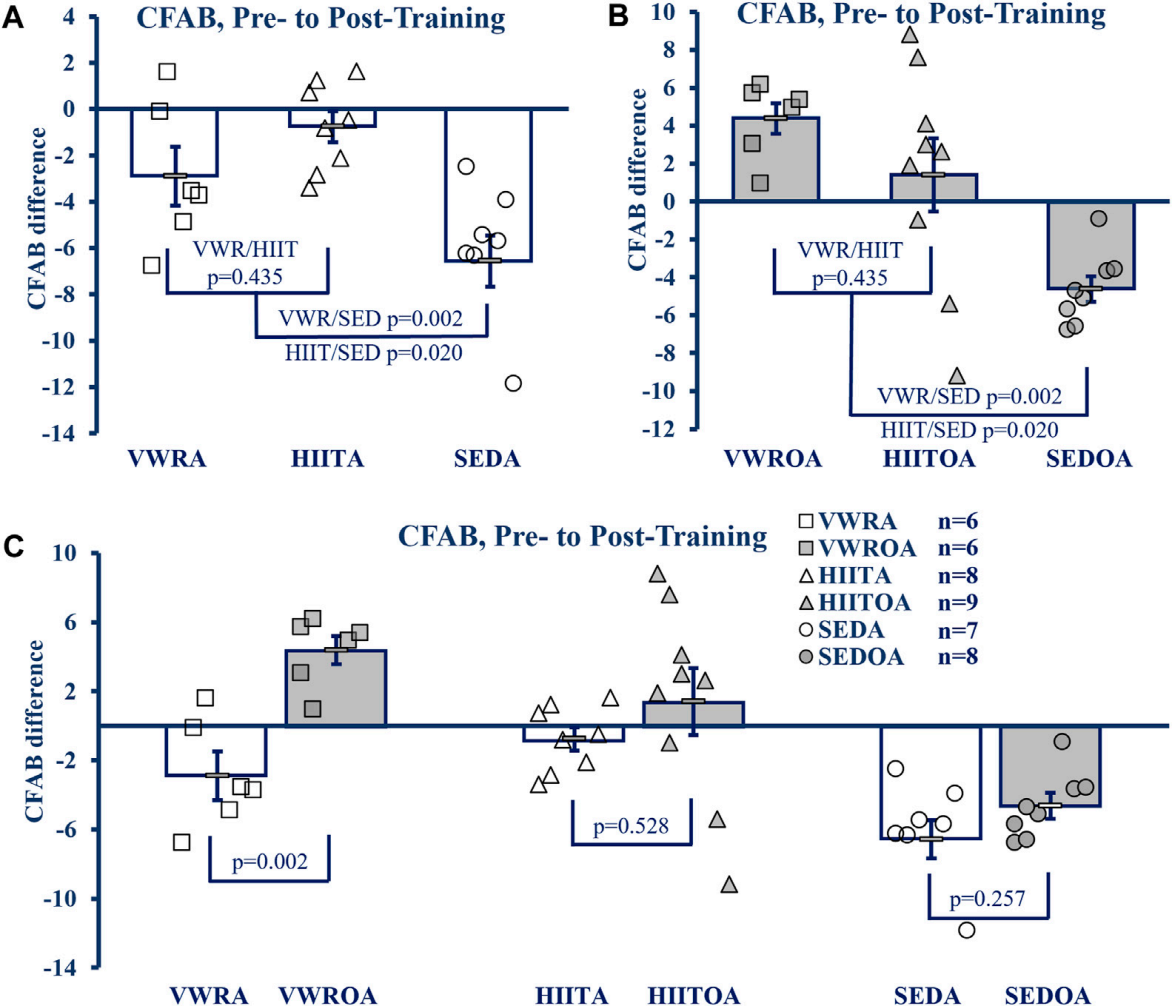
CFAB Difference Pre-to Post-Training. **(A)** Comparison of Exercise Groups in Adults **(B)** Comparison of Exercise Groups in Older Adults), **(C)** Comparison of Exercise Groups By Age. KEY: CFAB: comprehensive functional assessment battery composite scoring system. A more negative score means less functional ability and lower exercise capacity. Symbols: Each symbol (square, VWR; triangle, HIIT; or circle, SED) represents the change in CFAB score for one individual male mouse, while bars and error bars represent group mean and standard error. Ages and Groups: Open symbols = adult groups, shaded symbol = older adults. VWR = voluntary wheel running, HIIT = high intensity interval training, SED = sedentary control, A = adults (10 m at study end), OA = older adults (26 m at study end, except SEDOA = 28 m). *Statistical Tests:* Error bars = standard error, *p*-value (*p*), effect size η^2^ (partial eta squared) or Cohen’s d. Panels A) and B) One-Way ANOVA (panel A: F = 5.768 *p* = 0.012, η^2^ = 0.404; panel B: F = 8.38, *p* = 0.002, η^2^ = 0.456) with Tukey’s HSD (honest significant difference) *post hoc* testing, C) *p* from Student’s T-test (age comparison of exercise effects), effect sizes (Cohen’s d) = −2.324, −0.605, and −0.736 for VWR, HIIT, and SED respectively.

To determine exercise efficacy, and age-associated functional loss over the training period, we measured the difference between pre- and post-training CFAB values, producing an intervention assessment value similar to systems used previously (Graber, et al., 2015; Seldeen, et al., 2019). Exercise effects within age groups are shown in Figures 2A,B, while the effect of age on training is shown in Figure 2C.

In the adult groups the mean CFAB difference from pre-to post-training (One-Way ANOVA, F = 8.621, *p* = 0.002, η^2^ = 0.489, Tukey’s HSD *post hoc*) did not change with respect to training types (mean difference VWRA -2.873 ± 1.20, HIITA -0.842 ± 0.679, *p* = 0.341), but both training groups demonstrated preservation of function during aging compared to the sedentary control (SEDA mean difference −6.409 ± 1.126, *p* = 0.062 compared to VWRA and *p* = 0.002 compared to HIITA).

Similarly, in the older adult groups the mean CFAB difference from pre-to post-training (F = 7.394, *p* = 0.004, η^2^ = 0.425) were not different between exercise groups (VWROA 3.214 ± 0.919, HIITOA 0.499 ± 1.935; *p* = 0.426), but both groups again demonstrated significant preservation of function during aging compared to the sedentary control (SEDOA -4.837 ± 0.756, *p* = 0.004 compared to VWROA and *p* = 0.034 compared to HIITOA).

To compare the effect of VWR, HIIT and SED based on age, we evaluated the mean CFAB difference pre-to post-training of each exercise type using Student’s independent samples t-test. VWROA improved function compared to VWRA (t = 4.027, *p* = 0.002, Cohen’s d = 2.325). However, HIITA and HIITOA did not differ in effect between the ages (t = 0.654, *p* = 0.528, Cohen’s d = 0.302), and SEDA and SEDOA both declined similarly (t = −1.186, *p* = 0.257, Cohen’s d = −0.614).

We used a 3 × 2 × 2 mixed model ANOVA (3 exercise types, 2 ages, 2 time points, pre- and post-training) and compared CFAB measurements with-in subjects directly (F = 15.392, *p* < 0.001, partial η^2^ = 0.288) and both the main effects of exercise type collapsed across age (F = 12.917, *p* < 0.001, partial η^2^ = 0.408) and age collapsed across exercise type (F = 13.083, *p* < 0.001, partial η^2^ = 0.254) were significant. Between subjects the main effect of age collapsed across exercise was significant (F = 18.618, *p* < 0.001, partial η^2^ = 0.329, mean difference of −3.502 ± 0.812), though collapsing all exercise types across age was not significant (F = 0.367, *p* = 0.696, partial η^2^ = 0.019) and the interaction of age and exercise type tended toward a difference (F = 2.579, *p* = 0.089, partial η^2^ = 0.120).

Mouse performance on individual functional tests can reveal interesting trends in particular physical skills most affected by exercise. See Supplementary Table S1 for pre-training baseline and post-training means, and statistical analysis of the raw data including means testing, skew, kurtosis, and normality testing.

### Body composition: improved with exercise

**FIGURE 3:**
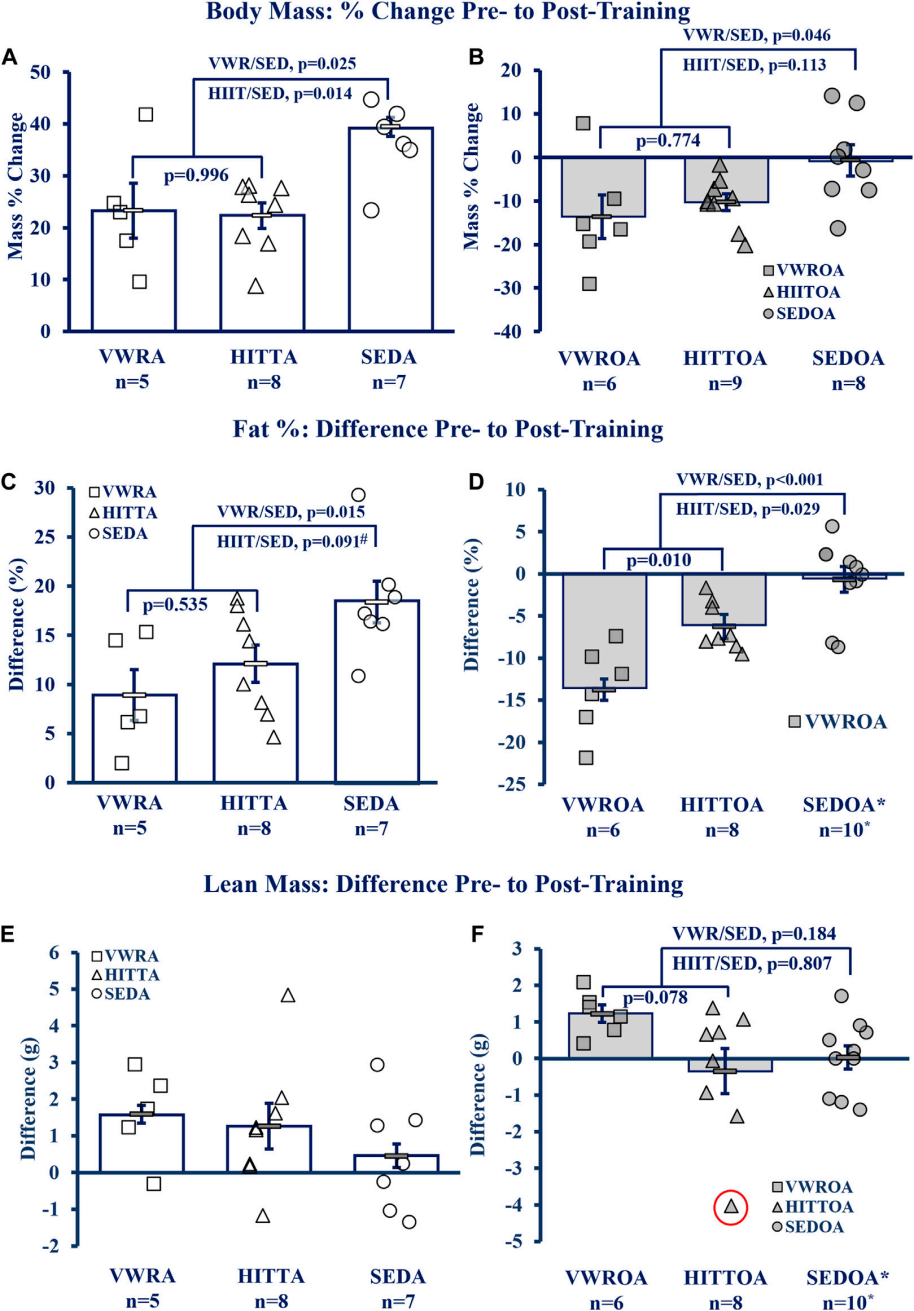
Body Composition Improved by Exercise. **(A, B)** Body Mass, **(C, D)** Fat percentage (Fat%), **(E, F)** Lean Mass Difference. KEY: Symbols: Each symbol (square, VWR; triangle, HIIT; or circle, SED) represents the measurement from MRI (magnetic resonance imaging) for one individual male mouse, while bars and error bars represent group mean and standard error. Ages and Groups: Open symbols = adult groups, shaded symbol = older adults. VWR = voluntary wheel running, HIIT = high intensity interval training, SED = sedentary control, A = adults (10 m at study end), OA = older adults (26 m at study end, except SEDOA = 28 m). g = grams. NOTE: *Fat% difference and lean mass change (from DEXA) in SEDOA taken from previously reported study (Graber et al., 2019b) for illustrative purposes only since we were unable to perform MRI on the SEDOA group due to Covid19 lab lockdowns. *Statistical Tests:* Means analysis by One-Way ANOVA with Tukey’s HSD (honest significant difference) *post hoc* testing: **(A)** F = 6.085, *p* = 0.010, η^2^ = 0.417, **(B)** F = 3.806, *p* = 0.040, η^2^ = 0.276, **(C)** F = 5.236, *p* = 0.016, η^2^ = 0.368, **(D)** F = 17.865, *p* < 0.001, η^2^ = 0.630, # trend (*p* = 0.091) between HITTA and SEDA, **(E)** no significant differences or trends: F = 1.196, *p* = 0.325, η^2^ = 0.117, **(F)** F = 2.810, *p* = 0.083, η^2^ = 0.211; Circled triangle = significant outlier; without outlier: F = 3.220, *p* = 0.061, η^2^ = 0.244, (no difference between VWROA and HIITOA, n = 7, *p* = 0.143; between VWROA and SEDOA, *p* = 0.060).

Exercise of either type improved the body composition outcomes of body mass percent change and the difference in pre-to post-training to similar degrees within age-groups, though lean mass difference was largely unchanged (see Figure 3A, B. Body Mass; Figure 3C, D Fat Percentage; Figure 3E, F Lean Mass). Adult exercise mice were resistant to body mass and fat% increases compared to the sedentary controls, whereas in the OA exercise mice both body mass and fat% were reduced compared to the SEDOA (note: values used for SEDOA fat% were taken from and earlier publication (Graber et al., 2019a) of a different population because we were unable to use MRI on the SEDOA mice due to the Covid19 lockdown). See Supplementary Table S2 for pre-training baseline and post-training means, and statistical analysis of the raw data including means testing, skew, kurtosis, and normality testing.

#### Body mass

Total body mass also altered between pre- and post-training (Figure 3A, B), except for the SEDOA which remained stable. Statistics below for the adult groups are from pair-wise comparisons from pre-to post-training from a 3 × 2 mixed model ANOVA within age groups (3 groups, VWR, HIIT< and SED; two time points, pre- and -post-training; with-in subject pair-wise comparisons). Body mass in all of the adult groups increased over the course of the experiment (with-in subjects effects, interaction of time and groups: F = 3.991, *p* = 0.037, partial η2 = 0.307). VWRA body mass increased 6.607 ± 1.179 g (*p* < 0.001), HIITA increased 6.594 ± 1.201 g (*p* < 0.001), and SEDA increased 10.376 ± 1.091 g (*p* < 0.001). The SEDA gained both overall mass and increased fat percentage by 18.40 points (pre-mean of 13.98% fat to 32.38% fat, post-mean) indicating that the SEDA mice gained primarily fat (mean difference pre-to post-training of 8.65 g ± 1.16 g) and a small amount of lean mass (average 0.46 g ± 0.67 g). Both older adult exercise groups lost mass. Older adult mass violated normality and *p*-values following are from the Wilcoxon Signed Rank test. The VWROA group lost an average of 6.01 ± 2.44 g of body mass (*p* = 0.046) while HIITOA lost 4.01 ± 0.71 g (*p* = 0.012). SEDOA mice did not gain weight and remained indistinguishable to their pre-training mass (mean mass change = −0.26 ± 2.44 g, *p* = 0.779).

#### Fat % and lean mass

Body composition (Fat%) altered markedly between the start and end of the experiment for all measured groups other than SEDOA (see Figures 3C,D). Statistics below for adult mice are from pair-wise comparisons from pre-to post-training using a 3 × 2 mixed model ANOVA interaction of exercise and groups (3 groups, VWR, HIIT and SED; two time points, pre- and -post-training; with-in subject pair-wise comparisons: F = 5.237, *p*=<0.001, partial η2 = 0.873). All adult groups gained body fat. VWRA, HIITA, and SEDA all increased body fat percentage: VWRA +8.97 ± 2.20% fat (*p* < 0.001), HIITA +12.14 ± 1.91% fat (*p* < 0.001), SEDA +18.40 ± 2.12% body fat (*p* < 0.001). The older adult mice had normality violations and the with-in subject tests for changes in fat% were performed using the Wilcoxon Signed Rank Test. Both VWROA and HIITOA mice lost body fat: VWROA -13.74% ± 5.03% body fat (*p* = 0.028), HIITOA -6.24% ± 2.88% body fat (*p* = 0.012). SEDOA mice in our prior study (Graber, et al., 2019) did not lose body fat (mass percent change −0.66% ± 1.43%, *p* = 0.953), for illustrative purposes the is data reproduced in Figure 3D. Thus, both exercise types mitigated increases in body fat associated with 6- to 10-month aging and decreased fat% in older adult mice.

Lean mass differences between groups were not significant, though there was a tendency (*p* = 0.078, Tukey’s HSD) for VWROA to increase lean mass compared to HIITOA (see Figure 3E, F, and Supplementary Table S2).

#### Muscle mass

**FIGURE 4:**
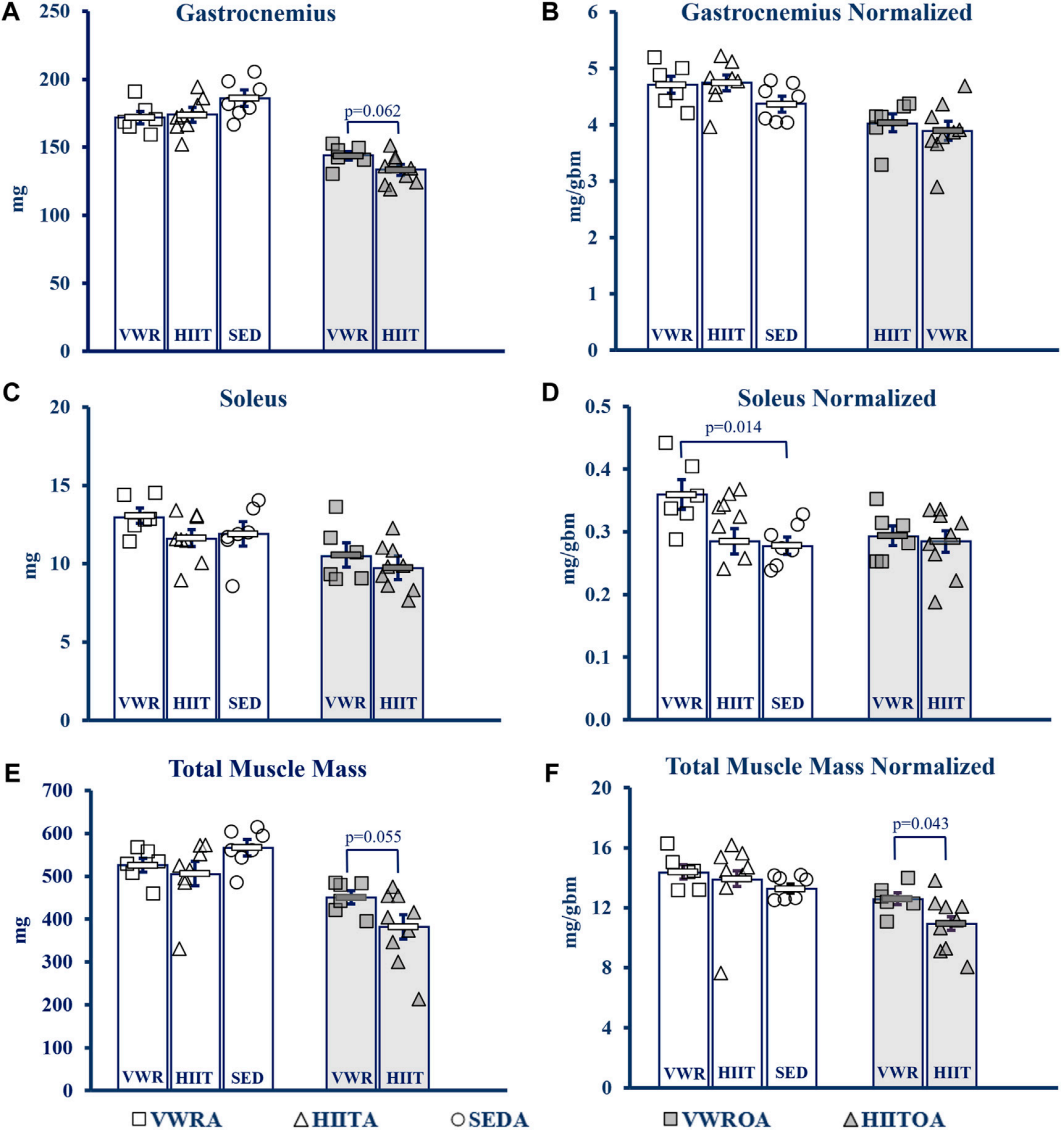
Muscle Mass Changes with Exercise. **(A)** Gastrocnemius (GAS). **(B)** GAS normalized to body mass. **(C)** Soleus (SOL). **(D)** SOL normalized to body mass. **(E)** Total muscle mass. Calculated by summing the mass of GAS, SOL, Plant, TA and Edl. **(F)** Total muscle mass normalized to body mass. KEY: Units: mg = milligrams, gbm = grams body mass. Symbols: Each symbol (square, VWR; triangle, HIIT; or circle, SED) represents the measurement for one individual male C57BL/6 mouse, while bars and error bars represent group mean and standard error. Ages and Groups: Open symbols = adult groups, shaded symbols = older adults. VWR = voluntary wheel running, HIIT = high intensity interval training, SED = sedentary control, A = adults (10 m at study end), OA = older adults (26 m at study end, except SEDOA = 28 m). VWRA, open squares (n = 7); HIITA, open triangles (n = 8); SEDA, open circles (n = 6); VWROA, shaded squares (n = 7); HIITOA, shaded triangles (n = 10). *Statistical Tests:* Means analysis by One-Way ANOVA with Tukey’s HSD (honest significant difference) *post hoc* testing, effect size η^2^ (partial eta squared for adult groups, effect size η^2^ (partial eta squared), and Student’s independent samples t-test for Older Adult groups: **(A)** F = 1.584, *p* = 0.232, η^2^ = 0.1; t = 2.042, *p* = 0.620, Cohen’s d = 1.076, **(B)** F = 1.584, *p* = 0.232, η^2^ = 0.150; t = 0.951, *p* = 0.359, Cohen’s d = 0.005, **(C)** F = 1.584, *p* = 0.232, η^2^ = 0.150; t = 0.951, *p* = 0.359, Cohen’s d = 0.501, **(D)** F = 5.037, *p* = 0.018, η^2^ = 0.359; t = 0.239, *p* = 0.815, Cohen’s d = 0.126, **(E)** F = 1.971, *p* = 0.168, η^2^ = 0.160; t = 2.14, *p* = 0.055, Cohen’s d = 0.984, **(F)** F = 0.479, *p* = 0.627, η2 = 0.051; t = 2.254, *p* = 0.043, Cohen’s d = 1.053.

Hindlimb muscles were collected, blotted dry, and weighed following euthanasia (see Figure 4). Statistics for data below from One-Way Univariate ANOVAs, and Student’s t-tests. NOTE: Muscles from SEDOA were not collected for this study and so values are only presented for the other five groups. See Supplementary Table S3 in the Supplement for raw data values of the tested muscles: gastrocnemius (GAS), plantaris (PLANT), soleus (SOL), tibialis anterior (TA), extensor digitorum longus (EDL), heart, and total muscle (sum of the other 5 hindlimb muscles) in mg and normalized to body mass including means testing, skew, kurtosis, and normality testing.

In the adult mice there was little overall change in muscle mass. Only SOL normalized to body (1-Way ANOVA, F = 5.037, *p* = 0.018, partial η^2^ = 0.359, Tukey’s HSD *post hoc*) mass demonstrated that the VWR increased mass compared to the SED (*p* = 0.014), but VWR was not different than HIIT. HIIT did not change compared to SED.

In the older adult mice, we only compared the two exercise types. VWR tended to improve muscle mass compared to HIIT in the total muscle mass measurement with a strong effect size (Student’s t-test, t = 2.134, *p* = 0.055, Cohen’s d = 0.974). This was confirmed when the total muscle mass was normalized to body mass (Student’s t-test, t = 2.254, *p* = 0.043, Cohen’s d = 1.053). GAS mass tended to be larger in VWR compared to HIIT, also with a strong effect size (Student’s t-test, t = 2.042, *p* = 0.062, Cohen’s d = 1.076), but this advantage was erased when normalized (Student’s t-test, t = 0.580, *p* = 0.572, Cohen’s d = 0.306).

### Plantar flexor peak tetanic torque (*in vivo* contractile physiology)

**FIGURE 5:**
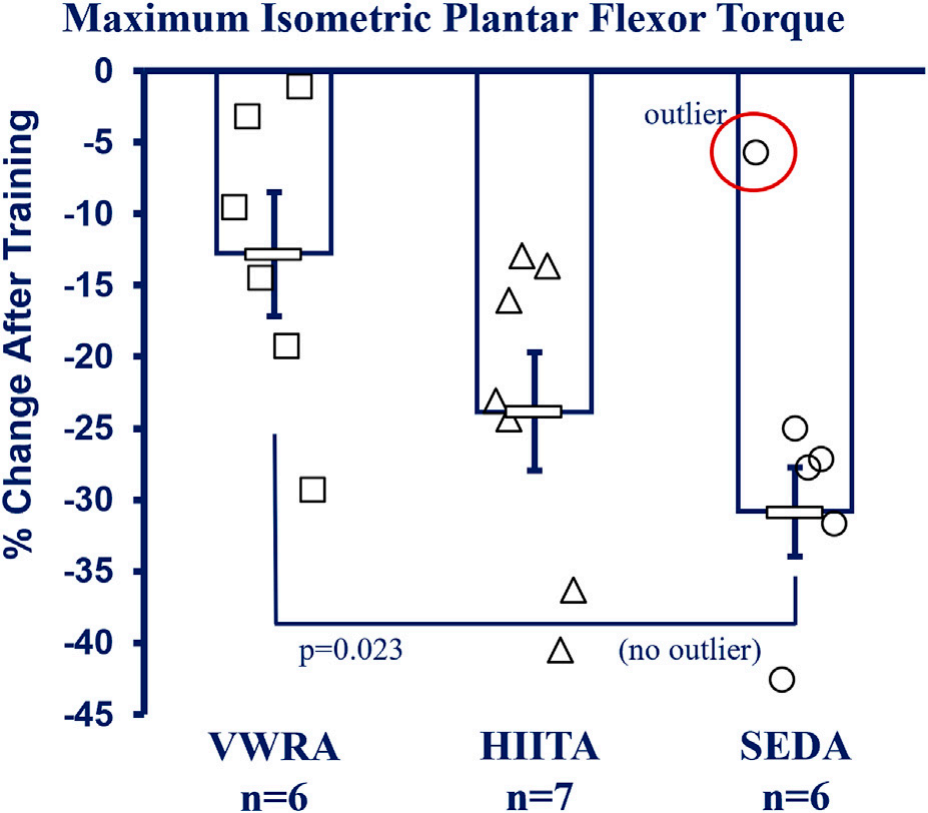
*In vivo* Contractile Physiology. All groups had decreases in maximum plantar flexor (gastrocnemius complex) isometric torque between pre- and post-training (means shown without the outlier in SED, −5.76% change >2.5 SD from the mean—marked as circled open circle symbol, NOTE: with outlier included there is no difference between groups). Maximum tetanic isometric torque was measured (mN*m) and is reported as percent change after correction to body mass (mN*m/gbm). KEY: Units: mN = milliNewtons, m = meters, gbm = grams of body mass. Symbols: Symbols: Each symbol (square, VWR; triangle, HIIT; or circle, SED) represents the measurement for one individual male C57BL/6 mouse, while bars and error bars represent group mean and standard error. Ages and Groups: Open symbols = adult groups (NOTE: older adults data not collected). VWR = voluntary wheel running, HIIT = high intensity interval training, SED = sedentary control, A = adults (10 m at study end). n:VWRA, open squares (n = 6); HIITA, open triangles (n = 7); SEDA, open circles (n = 6). *Statistical Test:* With SED outlier: no statistical difference between groups. 1-Way ANOVA with Tukey’s HSD (honest significant difference), Circled circle = significant outlier: F = 2.605, *p* = 0.105, η2 = 0.246. Without SED outlier: F = 4.704, *p* = 0.026, η2 = 0.385; VWRA vs. HITTA (*p* = 0.147), VWRA vs. SEDA (*p* = 0.023), HIITA vs. SEDA (*p* = 0.464).

See Figure 5 for more details and Supplementary Table S4 in the Supplement for raw data values including means testing, skew, kurtosis, and normality testing. NOTE: Because of technical limitations and the Covid19 global pandemic, we do not have data for the older adult groups (26 m). There was also a significant outlier in the SEDA group (−5.76% change from pre-to post, SD > 3 from the mean) that increased variability and we report both with and without the outlier (Figure 5 has the outlier designated, but the mean and statistics exclude it). With the outlier kept, there was no significant difference in the mean percent change (pre-to post-training) between groups using a one-way Univariate ANOVA (F = 2.605, *p* = 0.105, partial η2 = 0.246). However, without the outlier VWRA > HIITA > SEDA (F = 4.704, *p* = 0.026, partial η2 = 0.385), with Tukey’s HSD *post hoc* VWRA = HIITA (*p* = 0.147), VWRA > SEDA (*p* = 0.023), HIITA = SEDA (*p* = 0.464). Within the groups from pre-to post-training (3 × 2 ANOVA, F = 63.058, *p* < 0.001, partial η2 = 0.102) all the groups lost torque (normalized to body mass): VWRA -0.047 ± 0.018 mN*m/g (*p* = 0.018), HIITA -0.089 ± 0.016 mN*m/g (*p* < 0.001), when including the outlier SEDA decreased by 0.102 ± 0.018 mN*m/g (*p* < 0.001) and without outlier SEDA decreasing by 0.118 ± 0.009 mN*m/g (*p* < 0.003).

### Exercise volume

See the Online Only Supplemental Results section and Supplementary Figures S1, S2 for more details.

## Discussion

As we grow older, declining physical function is associated with loss of muscular strength, endurance, and mobility (McPhee et al., 2016; Nascimento et al., 2019). This decline leads to reduced ability to perform activities of daily living and a lower quality of life (McPhee et al., 2016; Marzetti et al., 2017). Consequently, older adults with greatly declined functional capacity lose independence and require assisted living (Marzetti et al., 2017). Exercise, while not a cure for sarcopenia, is a promising therapy to potentially retard the slope of age-related functional loss while improving quality of life. Exercise increases functional ability in older adults while also improving biomarkers of physical fitness such as strength, muscle mass, and VO_2max_ (Fiatorone et al., 1990; Bonnefoy et al., 2003; McPhee et al., 2016).

In this study we originally hypothesized that HIIT would prove to be a universally more efficacious exercise paradigm than VWR. The data supported partly supported our hypothesis in the adult 10 m male mice, but this did not hold true in the older 26 m male mice, which seemed to benefit equally from either type of exercise.

### Exercise: VWR or HIIT?

Our results complement existing literature findings that exercise improves or preserves measurements of health and function in both humans and rodents (Peterson et al., 2009; Garcia-Valles et al., 2013; Graber et al., 2015; Steffl et al., 2017; Feito et al., 2018; Seldeen et al., 2018; Ben-Zeev and Okun, 2021). In work from our lab and others, both VWR and HIIT protocols have demonstrated attenuated frailty and mitigated functional loss in mice (Graber et al., 2015; Gomez-Cabrera et al., 2017; Seldeen et al., 2018; Graber et al., 2019a; Seldeen et al., 2019; Gioscia-Ryan et al., 2021; Bisset et al., 2022). However, to date, there is little information in the literature regarding side-by-side comparisons of the efficacy of these two pre-clinical exercise paradigms, thus our current study focus.

### VWR

Voluntary wheel running in mice improves both body composition and physical function (Graber et al., 2015; Manzanares et al., 2019). VWR design is voluntary in nature--an individual mouse chooses their running volume and intensity. Most (but not all) mice will run on wheels, though the volume run varies widely between individuals. To ensure that mice randomized to the VWR group would run enough to be beneficial, we instituted an *a priori* exclusion criteria cut-off of <500 revolutions per week during pre-randomization CFAB testing. Mice excluded from the VWR trial were randomized into the HIITor SED groups. In retrospect, this may have unavoidably ensured the VWR group was home to more naturally active mice.

VWR is a common endurance exercise mouse model. Comparing VWR to a human exercise trial is problematic because the mice have no proscribed volume or intensity. A comparable human model to VWR would be one of tracking physical activity rates. Clinical studies incorporating self-reported activity levels indicate that, like the mice in this study, more frequent daily activity is associated with improved frailty scores (Manini et al., 2012; Savela et al., 2013; Tak et al., 2013). Thus, one might think of voluntary wheel running as translatable to a clinical trial where older adults are encouraged to start walking or jogging (tracked by pedometer), at a self-determined intensity and volume (Cheatham et al., 2018; Teixeira et al., 2021). Additionally, we must note that the size and type of wheels used influence the amount of running. Larger mice may run less on smaller in-cage style wheels designed for use in specific pathogen free environments, such as the ∼30 cm circumference wheels we use (Graber et al., 2021; Manzanares et al., 2019). This may partially account for why the 10 m mice in our study had a larger drop-off in VWR distance totals over time than we expected (with an average VWR group body mass gain of 22.7% from 6 to 10 months).

### HIIT

Both human and animal models of HIIT make use of standardized protocols accounting for the principles of exercise physiology such as progressive difficulty, intensity and volume. With mice, perceived exertion is detected by third party observation (how easily are the mice keeping up with the treadmill).

Seldeen et al. have previously shown that HIIT in 24-month-old mice resulted in improved frailty scores (Seldeen et al., 2018) and that long-term HIIT training (16 weeks) increased maximal treadmill time in older adult mice.

Prescribed high-intensity exercise has been previously shown to improve measures of frailty in elderly humans, including improvements in strength and flexibility (Hess et al., 2006; Buckinx et al., 2018; Losa-Reyna et al., 2019), stair climbing (Bonnefoy et al., 2003), walking time (Bonnefoy et al., 2003; Losa-Reyna et al., 2019), chair rising (Bonnefoy et al., 2003; Buckinx et al., 2018), and hand grip strength (Jimenez-Garcia et al., 2019). Additionally, high-intensity exercise in humans has long been shown to improve markers of frailty and sarcopenia, including oxygen consumption, cardiovascular function, blood pressure, and inflammation (Fiatorone et al., 1990; Bonnefoy et al., 2003; McPhee et al., 2016; Buckinx et al., 2018).

One of the hallmarks often touted as a benefit of HIIT for humans is the time-saving nature—more exercise for equal or better benefit in a shorter time period than traditional long distance endurance training. This holds true for mice as well. In our study the adult mice eventually worked up to a 3x/week training period of 6–10 min of intense activity with 6–7 min of lower intensity recovery/warm-up/cool-down time per exercise bout; with the older adult mice having a little less. The VWR mice, on the other hand, spread out their exercise bouts over multiple time periods accounting for a greater expenditure of time during their 4 days per week of training in that their average time spent running on the wheel (which is measured in 10-min increments) account for 13–17 h total. Please see Supplementary Figures S1, S2 in the Supplement for more details.

From the standpoint of amount of time spent exercising in comparison to benefits gained by attenuating functional loss, HIIT clearly shows superiority to VWR. However, maximal benefit in the older mice is less clearly defined, and VWR is equivalent to HIIT (albeit with a greater time investment). However, HIIT exercise significantly increased older adult work-out intensity compared from pre-to post-training, while VWR does not (see Supplementary Figure S1, S2). Overall, though, functional benefits are similar, contrary to our original primary hypothesis. We now suggest that endurance exercise training of any type improves or preserves physical function in untrained older adult male mice, although based on our results from the adult groups, HIIT may better mitigate functional loss in early middle age male mice (see Supplementary Figure S1B, C; Supplementary Figure S2D).

Both older adult groups maintained or improved physical function following exercise training. There was wide variation in individual results, suggesting that for older adults mice, after a lifetime of sedentary living, starting any program of physical activity successfully prevented functional loss, though, for any given individual, the specific type of exercise may matter. In contrast, all adult groups decreased overall function. These mice aged from young adult males (6-months old) to early middle-age males (10-months old) during the study. The onset of middle age is associated with reduced physical function and reduced physical function in mice aging from 6-10 months has been reported previously (Yanai and Endo, 2021). However, the pre-to post-training decrease in exercise capacity (CFAB) shown by both adult exercise groups was significantly mitigated compared to the sedentary mice, indicating that early adoption of a lifestyle including regular exercise may slow age-related deterioration of physical function.

### Age and exercise

Contrary to our hypothesis, HIIT did not universally improve outcomes compared to VWR. The effects of the exercise types depended, in part, on the age of the exercising group (exercise type * age interaction). Both groups of older adult exercise mice had similar improvements in overall motor function (CFAB change, Figure 2), and body composition (Figure 3). The older exercisers differed in grip strength, with the VWR mice having improved forelimb strength (Supplementary Table S1), a surprising result potentially related to the running wheels having small ridges that the mice might grip with their front feet as they run. In addition, as we expected, the HIIT mice greatly increased their treadmill capability compared to the VWR group. Notably, total muscle mass and normalized SOL mass in both older adult exercise groups were similar to SEDA—suggesting an age-reversing rescue effect from exercise. This occurred most strikingly in the soleus, a postural and locomotive muscle with a large percentage of type 1 muscle fibers, which, of the muscles we measured, should respond the most to endurance training (Graber et al., 2015; Graber et al., 2023).

The results suggest that for older age sedentary individuals, any type of physical activity will result in improved functional performance; however, what happens after the initial activity increase overcomes the effect of detraining remains to be investigated. Commonly, “any exercise is better than no exercise” is a mantra used to encourage exercise participation by sedentary older adults, and the current study has demonstrated this in the context of older mice.

In contrast, compared to VWRA the HIITA benefited in rotarod, treadmill, and mitigated loss of overall function (CFAB). However, as with the older mice; VWRA had significantly stronger forelimbs than HIITA. Notably, exercise types did not differ in their effect on adult body composition.

Overall, while adult male mice all experienced a decrease in physical function between pre- and post-testing, older adult male exercise groups maintained performance. This may be because prior to entering the study at 22 months of age, both HIITOA and VWROA were sedentary (detrained) for their entire lives, potentially leaving them with more room for improvement compared to the adult mice at their 6-month prime-of-life point. It would be enlightening to follow a HIIT exercise group during a lifespan longitudinal study to observe how HIIT effects functional abilities, or whether training for a longer period (6 m or more) in older adult mice would demonstrate greater efficacy as the mice became more highly trained. There have been a number of long-term exercise trials in mice that determined VWR improved healthspan including physical and cognitive function (Garcia-Valles et al., 2013; Robison et al., 2018; Gioscia-Ryan et al., 2021). Retrospective studies in humans of master endurance athlete humans have shown that maintaining an endurance exercise program over time is essential to preserving cardiorespiratory fitness, since rapid linear degradation of VO_2max_ begins even after a few days of detraining whether in highly trained athletes or not (Burtscher, et al., 2022). Lifelong master athletes retain lean mass compared to less active older adults (Walker et al., 2023). Increased activity rate itself in humans is also linked to a plethora of positive outcomes, and both intense exercise as well as moderate activity promotes retention of muscle and physical function (Crane et al., 2013; Steffl et al., 2017). However, likely due to the labor-intensive aspects, randomized controlled trials of lifespan exercise with treadmill running, whether moderate or high-intensity, are understudied in both humans and rodents.

### Caveats

One major caveat is because of restrictions related to the SARS-CoV-2 global pandemic and the ensuing laboratory shut-downs, we were unable to supervise a 26 m SEDOA group or perform *in vivo* contractile physiology on the 26 m exercise groups. We did include data on a slightly older (age 24m–28 m) sedentary group as a substitute SEDOA, demonstrating an overall loss of function over a four-month time period that was not observed in either exercise group. There is also data in the literature demonstrating the increased functional capacity of mice exercised with both HIIT and VWR compared to age-matched controls (Graber et al., 2015; Seldeen et al., 2018; Seldeen et al., 2019).

Secondly, we may have erred on the side of caution in advancing the HIIT protocol intensity in the older mice. It is possible that we did not stimulate them to the highest degree possible as we were concerned about injuries. As we based the HIIT program for the adult mice to match that of the older mice, the amount of stimulation may have been suboptimal to ensure maximum exercise adaptations. In future work, a dose-response study is warranted to determine if greater intensity would increase gains. Never-the-less, it was clear from the study that exercise either improved or preserved functional performance relative to the sedentary control groups.

Third, as we have addressed previously (Graber et al., 2021), the small (30 cm circumference) VWR wheels designed to fit inside home cages and permissible in specific pathogen free facilities may encourage less running by larger mice who may find the wheels smaller than would be optimal. This results in fewer overall revolutions, which may be of less import when the goal is to measure volitional exercise/activity rate than if the VWR is being utilized as an exercise protocol. Thus, a major consideration when designing future exercise should be the size/type of running wheels and the sizes of the mice.

Finally, this study includes only information on male C57BL/6 mice. Our major outcome measure CFAB has to date only been validated in male mice. Future work should address potential sexual dimorphisms in exercise response, and any differences in other strains of mice.

## Conclusion

As the world population ages, it will become increasingly necessary to determine and evaluate therapeutic strategies to maintain physical function and independence for as long as possible. Exercise of all types is a critical component of this, but more information is needed to understand exactly how different exercise modalities affect functional capacity, sarcopenia and frailty. Eventually uncovering exercise mimetics may help provide these positive effects in individuals who are unable to participate in a standard exercise program (e.g., older adults in the hospital restricted to bedrest can lose 1 kg of lean leg muscle in just a week—which is very hard to regain) (Galvan et al., 2016). Furthermore, more research is needed on how to improve exercise response in older adults.

We have shown that exercise improves or preserves overall physical function, or mitigates functional loss, in both adult and older adult male C57BL/6 mice, but the pattern of improvement differs according to age. Younger adult male mice benefit more from high-intensity interval training while older adult male mice receive similar functional benefits from either high-intensity exercise or lower-intensity voluntary exercise. Thus, to extrapolate to humans, for untrained sedentary older male adults, getting started in any type of exercise program will improve physical function, though whether exercise type does influence adaptations after the initial training period (i.e., after counteracting the effects of detraining) remains to be investigated. Importantly, HIIT provided similar benefits to older male mice as VWR with a vastly reduced time commitment for exercise. This is a critical point when considering how best to structure exercise programs for individuals who may have multiple time constraints or limits on participation.

## Data Availability

The original contributions presented in the study are included in the article/Supplementary Material, further inquiries can be directed to the corresponding author.
