# Supplementary material for: Endurance exercise preserves physical function in adult and older male C57BL/6 mice: high intensity interval training (HIIT) versus voluntary wheel running (VWR)
Source: Front Aging. 2024 Mar 7;5:1356954. doi: 10.3389/fragi.2024.1356954 (PMC10958787; doi:10.3389/fragi.2024.1356954)
Supplement: Supplementary file 1 [file DataSheet1.PDF]

Appendix A: Supplemental Material

Endurance Exercise Preserves Physical Function in Adult and Older Male C57BL/6 Mice: High Intensity Interval Training (HIIT) versus Voluntary Wheel Running (VWR)

Megan L. Pajski[1], Chris Byrd[1], Nainika Nandigama[2], Emily Seguin[3], Anna Seguin[3], Alyssa Fennell[3], Ted G. Graber[#,1,3,4,5]

Affiliations: East Carolina University: [1]Dept. of Physical Therapy, [2] Dept. of Public Health, [3]Dept. of Kinesiology, [4] Dept. of Physiology, [5] East Carolina Obesity and Diabetes Institute

# Corresponding author: Ted G. Graber, email: grabert19@ecu.edu

Table of Contents

Content	Pages
Supplemental Methods	2
Supplemental Results	3
Supplemental Figure S1	4
Supplemental Figure S2	5
Supplemental Table S1-4	See Excel File
Supplemental Discussion	6
Supplemental Table S5	6
Supplemental References	8

Supplemental Methods

Functional Test Determinants of CFAB: (detailed protocols have been previously published at: Graber et al., 2021; Graber et al., 2019a; Graber et al., 2018; Graber et al., 2015; and Graber et al., 2013)

Rotarod:

The male C57BL/6 mice were acclimated over two days to use a Panlab LE820 rotarod (Harvard Apparatus). On the third day, the rotarod was set to start at a speed of 4 rpm and accelerate over a total period of 300 s. Latency to fall was recorded for each mouse and the test repeated for a total of three trials, with a minimum fifteen-minute rest between tests. The longest latency period was used for CFAB score determination.

Grip Meter:

To measure forelimb strength, mice gripped a grip strength apparatus (BioSeb GT3) set up with a grip bar. Mice were pulled straight backwards by their tail at a gentle, steady rate until they let go. Grip strength was recorded in newtons and the test was repeated for five total measurements. The highest grip force was used for CFAB score determination.

Treadmill:

Mice were trained over two days to use a treadmill (Columbus Instruments Exer 3/6) for a target minimum of two minutes per session. The treadmill shock grid was set to administer shocks at a repetition rate of 2 Hz, for a maximum of six shocks or three visits to the shock grid. On the third day, starting at a speed of 3 m/min and accelerating 0.6 m/min every 20 s, the time for each mouse to max out shocks was recorded, as well as the speed at shock-out. The time to shock-out was used for CFAB determination.

VWR (voluntary wheel running):

Each mouse was placed in a cage equipped with a running wheel (Columbus Instruments Mouse Home Cage Running Wheel) for seven days. Each running wheel was attached to a magnetic revolution counter. At the end of the seven days, total wheel turns were recorded and converted to km/day for CFAB score determination.

Inverted Cling:

Mice were placed in a custom built device consisting of a hinged grid top over a 56 cm tall x 29 cm wide x 27 cm deep plexiglass box with a padded bottom to determine four-limb strength and endurance, and the latency to fall was recorded. After a minimum fifteen-minute rest, the test repeated, and the maximum used for CFAB score determination.

Supplemental Results

Exercise Volume

See Supplemental **Figures S2** and **S3** for more details. Statistics below are from unpaired or paired Student's t-tests, as appropriate. Average work performed each week (grams of body mass, measured each week, multiplied by distance ran) for each male mouse was calculated for both VWR and HIIT. VWR started out trending (p=0.073) 10m to roughly 6.5 fold greater running distance than 26m (in both km/day and work, g*km/day), but, over time, the 10m VWR mice worked numerically less and less, from an overall average of 34.898 g*km/day in week 1 to 14.575 g*km/day at the end of the study at week 14, whereas the 26m VWR mice trending (p=0.075) to work more, from an average of 7.039 g*km/day in week 1 to 15.222 g*km/day average at the end of the study)—to essentially equivalent to the 10m mean (p=0.928). **Supplemental Figure S3** compares early-training (week 2, giving mice a week to adjust to the presence of the wheel) to late-training (week 13). The total number of active intervals (10-minute periods during which mice used the wheel) increased significantly for the VWRA (p=0.017), however, power output during these active intervals showed trended lower (p=0.076) from an initial average of 23.278 g*m/min to a late-training average of 8.859 g*m/min. VWROA demonstrated no significant change in either number of active intervals or power output over the course of training; however, early in training the power output by VWROA was significantly lower (p=0.047) than VWRA and this difference disappeared by late training. This indicates that the similar performance by both age groups by the end of training was due to a combination of reduced power output by maturing adult mice and maintenance of power output by older adult mice.

In contrast, the work done by the HIITA group was consistently and significantly more than the HIITOA group (2.22 kg*m 10m versus 0.77 kg*m 26m at week one to 4.48 kg*m 10m versus 2.78 kg*m 26m at week 12, **Figure S2B**), though the power production was greater in 10m initially (p<0.001), it was similar from week 7 on (3.31 g*m/s 10m versus 2.17 g*m/s 26m at week one to 3.99 g*m/s 10m versus 3.33 g*m/s 26m at week 12 (p=0.280), **Figure S2C**). Both HIIT exercise volume and power increased statistically and steadily in both age groups (work: between week 1 and 12, 10m p<0.001, 26m p<0.001; power: 10m p=0.008, 26m p<0.001). Overall, the 10m mice total mean work was 64% higher than 26m (3.51 kg*m 10m versus 2.14 kg*m 26m, p<0.001) though total power output between the two HIIT age groups was not statistically different, indicating volume of exercise was higher in HIITA but intensity of exercise was similar. Unlike VWR groups, both 10m (p=0.006) and 26m (p=0.010) showed a significant increase in power output between early and late training (**Figure S3D**), though only the 26m group showed a significant increase in running speed (p=0.019).

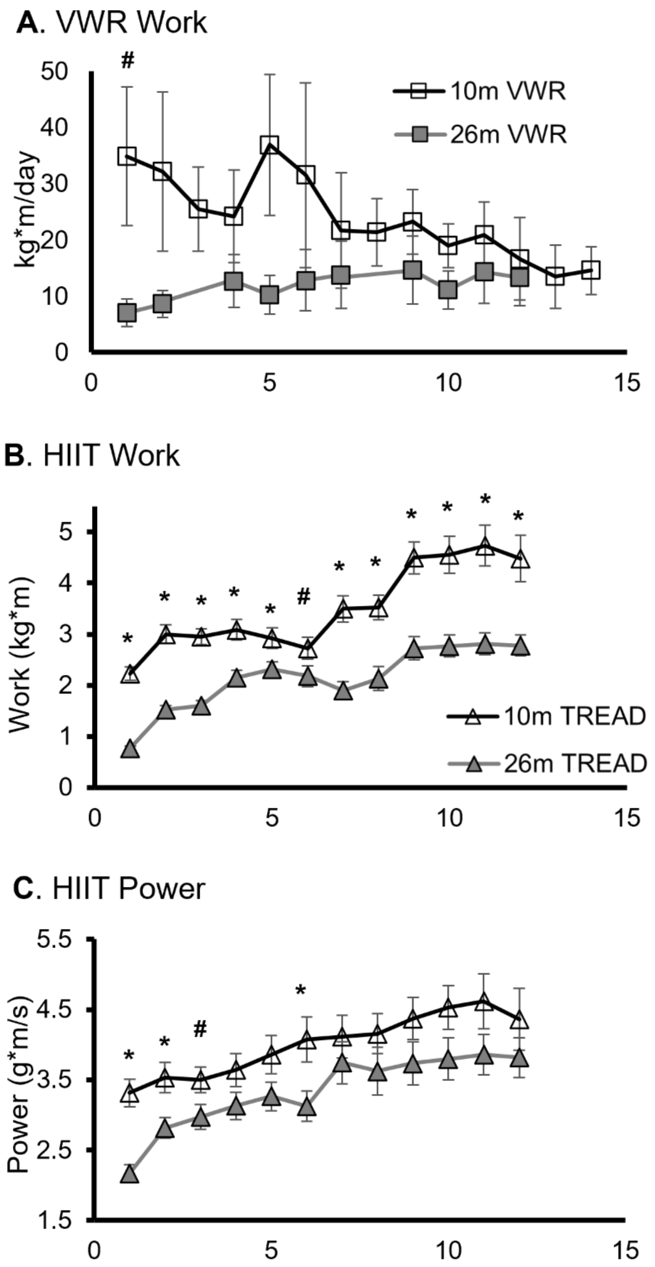

Figure S1 Exercise Volume per Week of Training. Progress in male mouse performance during exercise training. A) Overall work performed per week of VWR, data reported in kilograms of body mass times meters run per day (kg*m/day). B) Overall work performed per week of HIIT training, data reported in kilograms of body mass times total meters run (kg*m). C) Overall power produced during each week of HIIT training, data reported in grams of body mass times total meters run per second (g*m/s). 10m VWR, open squares (n=7); 26m VWR, shaded squares (n=7); 10m HIIT, open triangles (n=8); 26m HIIT, shaded triangles (n=10). Each symbol represents the group mean, while error bars represent standard error of the mean. Significance is represented by pound sign (#, $0.05<p<0.1$) or asterisk (*, $p<0.05$), calculated using a student's t test comparing group-and week-matched age groups.

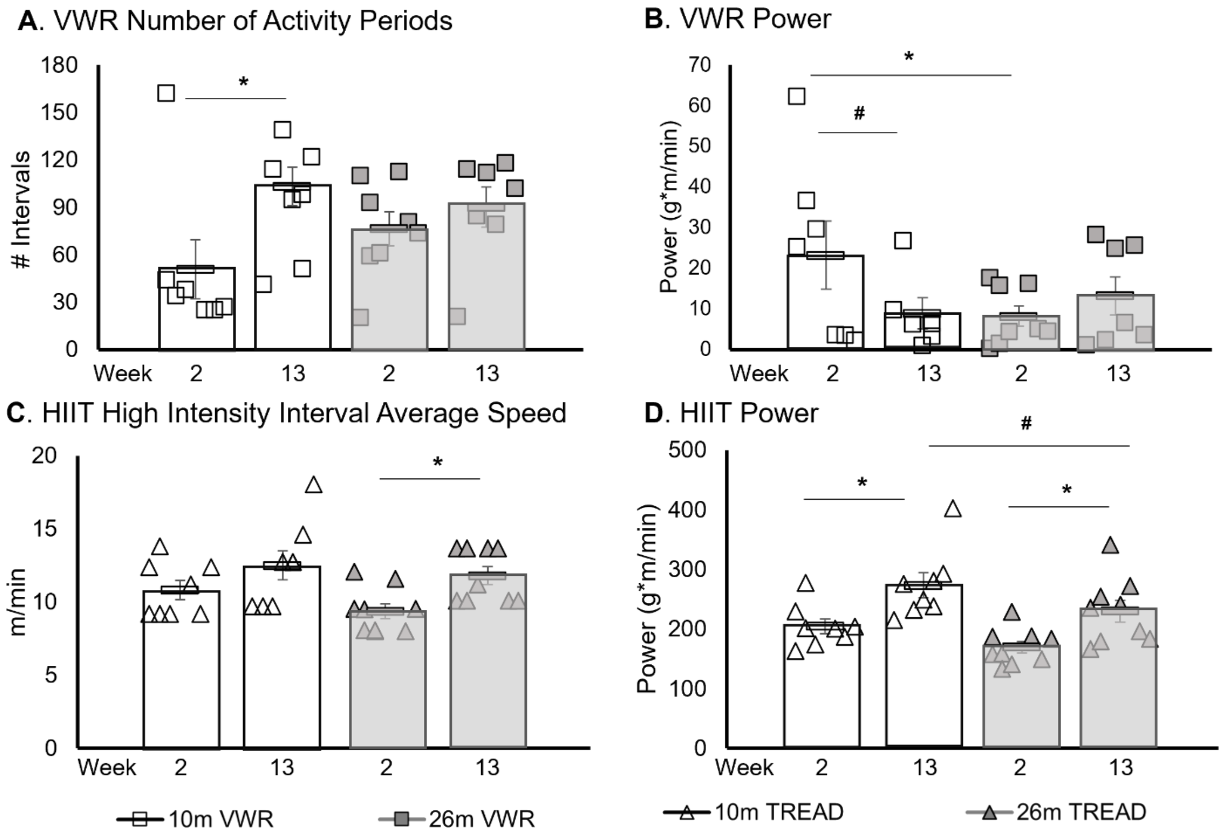

Figure S2 Comparison of VWR and HIIT Exercise Effects. Comparison of week 2 (early training) and week 13 (late training) male mouse performance during exercise training. **A)** Total number of activity periods during VWR. Intervals were recorded every 10 minutes, so the total number of activity periods is the number of intervals during which wheel revolutions were recorded (# intervals). **B)** Power produced per minute by VWR mice during activity periods, data reported in grams of body mass times meters run per minute (g*m/min). **C)** Average high intensity running speed during HIIT, data reported in meters per minute (m/min). **D)** Power produced during HIIT training, data reported in grams of body mass times total meters run per minute (g*m/min). **KEY**: 10m VWR, open squares (n=7); 26m VWR, shaded squares (n=7); 10m HIIT, open triangles (n=8); 26m HIIT, shaded triangles (n=10). Each symbol represents one individual mouse, while bars and error bars represent average and standard error of the mean for each group. Significance is represented by pound sign (#, $0.05<p<0.1$) or asterisk (*, $p<0.05$), calculated using a one-way univariate ANOVA with least significant differences post-hoc testing.

Online Only Supplemental Discussion Section

C57BL/6 Mouse Age Compared to Human Age

In male C57BL/6 percent survival is 90%, 75%, 50%, 25% and 10%, respectively at 18m, 24m, 27m, 30m, and 32m (https://www.nia.nih.gov/research/dab/aged-rodent-colonies-handbook/strain-survival-information). Life expectancy (mean lifespan) in male humans in the United states in 2020 was 74.2 years of age, or 890.4m and approximate survival rates in 2019 of human males were 89% (55y, years), 80% (65y), 73% (70y), 64% (75), 52% (80y), 36% (85y), 19% (90y) and 6% (95y).(Xu, et al., 2022; Murphy et al., 2021) Thus, based on survival rates, in older mice we roughly equate an 18m male C57BL/6 mouse with a 55 year old male human, a 24m mouse = 69y human, a 27m mouse = 79y human, 30m mouse = 86y human and a 32m mouse = 93y human. A rough estimate based only on mean male lifespan of 890.4m humans divided by 27m in mice equals 33 human months to mouse-for simplicity we will round it up to 1 human year = 3 months in mice (see **Table S3** for more details). Using this method, we estimate 22m mice to be the equivalent of 63y humans and 26m mice to be equivalent to 75y humans.

We estimate the ages of 6m and 10m old mice to be roughly 18y and 30y in humans based upon our 3m/y simplification. However, since mice and humans have quite different developmental ages and stages, estimating the age of mice at 100% survival is more problematic. Catherine Hagan at the Jackson Laboratories, based upon a cohort of 300 mice (50% male and 50% female), estimates C57BL/6 to mature 150 times human rates during the first month and then 45x humans for the next 5 months: adults at 3-6months (humans at 20-30y), middle-aged at 10-15m (humans from 38-47) and older adult mice as 18-24m (humans 56-69) for general purposes, *yet emphasize that depending upon the systems being studied these ages may need adjustment* (https://www.jax.org/news-and-insights/jax-blog/2017/november/when-are-mice-considered-old; https://www.jax.org/research-and-faculty/research-labs/the-harrison-lab/gerontology/life-span-as-a-biomarker). Similarly, one can use the method proposed by Dutta and Sengupta, estimating over the entire lifespan that 9.125 mouse days = 1 year, with nuances at various life stages—although they underestimated the lifespan of mice at 24m in their calculations of senescent mice since many mice routinely live much longer (see the first sentence). (Dutta and Sengupta, 2016) Because male mice are sexually mature at the age of 10-12 weeks but long bone growth is still active at 3 months (in male mice epiphyseal plates close by 4.5m) and in humans long bone growth ceases on average between 14 and 19 in humans (16.5 year average = 4 m in mice), for the purposes of our studies we consider full-grown adults to be starting at 5 months of age, when we are sure that no more long bone growth will occur and thus a 6 month old mouse we considered to be roughly equivalent to early-mid-20’s in human ages.

Older Adults	Males		Calc		Mouse Age (m)	Human Age (y)	M-Y Ratio	per m
Survival %	C57BL/6 Mice (m)[1]	Humans (y)[2]	m to y	y/m	2	13	6.50	
90	18	56	56	3.1	4	18	4.50	2.5
75	24	69	72	2.9	6	26	4.33	4
50	27	79	81	2.9	10	40	4.00	3.5
25	30	86	90	2.9	17	54	3.18	2
10	32	93	96	2.9	18	55	3.06	1
			mean	2.9	20	61	3.05	
Adolescent to Adult					22	65	2.95	
Sexual Maturity (mean)[3,4]	2	13		6.5	24	69	2.88	
long bone epiphyseal plate close (mean)[5,6]	4	18		4.5	26	75	2.88	
			mean	5.5				
Adult to Middle-Age								
Adult(5-9m, 18-39h)	4	18		4.5	27	79	2.93	
	9	39		4.3	28	82	2.93	
Adult-Early Middle Age	10	40		4.0	30	86	2.87	
Early-Late Middle Age	17	55		3.2	32	93	2.91	
			mean	4.0				

Table S5Calculations for Mouse Aging

References (all web pages accessed on 07/28/23 via WWW)

1 https://www.nia.nih.gov/research/dab/aged-rodent-colonies-handbook/animal-information
https://www.jax.org/research-and-faculty/research-labs/the-harrison-lab/gerontology/life-span-as-a-biomarker

2 https://www.cdc.gov/nchs/products/life_tables.htm#life
https://www.ssa.gov/policy/docs/rsnotes/rsn2016-02.html

3 Duta and Sengupta, 2016.

4 https://www.msdmanuals.com/en-gb/professional/pediatrics/growth-and-development/physical-growth-and-sexual-maturation-of-adolescents

5 Long bone growth in mice continues well past sexual maturity (Sher, et al., 2012; Kilborn, et al., 2002)

6 https://www.hopkinsallchildrens.org/Patients-Families/Health-Library/HealthDocNew/Growth-Plates
https://courses.lumenlearning.com/suny-ap1/chapter/bone-formation-and-development/

KEY: m=months of age, y=years of age, calc-conversion of months to years, y/m= years divided by months

References

1. Dutta S, Sengupta P. Men and mice: Relating their ages. Life Sci. 2016;152:244-248. doi:10.1016/j.lfs.2015.10.025
2. Graber TG, Maroto R, Fry CS, Brightwell CR, Rasmussen BB. Measuring exercise capacity and physical function assessment in adult and older mice. J Gerontol A Biol Sci Med Sci. 2021;76(5):819-824. doi: 10.1093/gerona/glaa205
3. Graber TG, Fandrey KR, Thompson LV. Novel individualized power training protocol preserves physical function in adult and older mice. Geroscience. 2019a. Apr;41(2):165-183. doi: 10.1007/s11357-019-00069-z. Epub 2019 May 10.
4. Graber TG, Rawls BL, Tian B, Durham WJ, Brightwell CR, Brasier AR, Rasmussen BB, Fry CS. Repetitive TLR-3-mediated Lung Damage Induces Skeletal Muscle Adaptations and Cachexia. Exp Gerontol. 2018;pii: S0531-5565(17)30667-8. doi: 10.1016/j.exger.2018.02.002.
5. Graber TG, Ferguson-Stegall L, Liu H, Thompson LV. Voluntary aerobic exercise reverses frailty in old mice. J Gerontol A Biol Sci Med Sci. 2015;70(9):1045–1058. doi: 10.1093/gerona/glu163
6. Graber TG, Ferguson-Stegall L, Kim J-H, Thompson LV. C57BL/6 Neuromuscular Healthspan Scoring System. J Gerontol A Biol Sci Med Sci. 2013;68(11): 1326-1336. doi: 10.1093/gerona/glt032
7. Kilborn SH, Trudel G, Uhthoff H. Review of growth plate closure compared with age at sexual maturity and lifespan in laboratory animals. Contemp Top Lab Anim Sci. 2002 Sep;41(5):21-6. PMID: 12213043
8. Murphy SL, Kochanek KD, Xu J, Arias E. Mortality in the United States, 2020. *NCHS Data Brief*. 2021;(427):1-8.
9. Sher RB, Cox GA, Ackert-Bicknell C. “Chapter 2.5 Development and Disease of Mouse Muscular and Skeletal Systems” from: The laboratory Mouse, 2nd Ed., Ed Hedrich HJ; Academic Press, Elsevier Amsterdam, 2012, pg 230.
10. Xu J, Murphy SL, Kochanek KD, Arias E. Mortality in the United States, 2021. *NCHS Data Brief*. 2022;(456):1-8
